# Mutant KRAS drives metabolic reprogramming and autophagic flux in premalignant pancreatic cells

**DOI:** 10.1038/s41417-021-00326-4

**Published:** 2021-04-08

**Authors:** Tatsunori Suzuki, Takahiro Kishikawa, Tatsuyuki Sato, Norihiko Takeda, Yuki Sugiura, Takahiro Seimiya, Kazuma Sekiba, Motoko Ohno, Takuma Iwata, Rei Ishibashi, Motoyuki Otsuka, Kazuhiko Koike

**Affiliations:** 1 Department of Gastroenterology, Graduate School of Medicine, The University of Tokyo, Tokyo, 113-8655 Japan; 2 Department of Cardiovascular Medicine, Graduate School of Medicine, The University of Tokyo, Tokyo, 113-8655 Japan; 3 Department of Biochemistry, Keio University School of Medicine, Tokyo, 160-8582 Japan

**Keywords:** Pancreatic cancer, Cell biology

## Abstract

Mutational activation of the *KRAS* gene occurs in almost all pancreatic ductal adenocarcinoma (PDAC) and is the earliest molecular event in their carcinogenesis. Evidence has accumulated of the metabolic reprogramming in PDAC, such as amino acid homeostasis and autophagic flux. However, the biological effects of *KRAS* mutation on metabolic reprogramming at the earlier stages of PDAC carcinogenesis are unclear. Here we report dynamic metabolic reprogramming in immortalized human non-cancerous pancreatic ductal epithelial cells, in which a *KRAS* mutation was induced by gene-editing, which may mimic early pancreatic carcinogenesis. Similar to the cases of PDAC, *KRAS* gene mutation increased the dependency on glucose and glutamine for maintaining the intracellular redox balance. In addition, the intracellular levels of amino acids were significantly decreased because of active protein synthesis, and the cells required greater autophagic flux to maintain their viability. The lysosomal inhibitor chloroquine significantly inhibited cell proliferation. Therefore, metabolic reprogramming is an early event in carcinogenesis initiated by *KRAS* gene mutation, suggesting a rationale for the development of nutritional interventions that suppress or delay the development of PDAC.

## Introduction

Pancreatic ductal adenocarcinoma (PDAC) is the fourth leading cause of cancer-related death in the United States [1]. The 5-year survival rate of patients with PDAC is 9% [1] and it is predicted that PDAC will, in the near future, become the second leading cause of cancer-related death [2]. The development of interventions that interrupt carcinogenesis will require an understanding of the early biological events during PDAC carcinogenesis.

The acquisition of a gain-of-function mutation in *KRAS* is the predominant driver of malignant transformation in various tissues [3]. Notably, more than 90% of PDAC cases have *KRAS* mutations [4–8], and approximately 50% of colorectal cancer has mutations in *RAS* or a *RAS*-related pathway [9]. The development of PDAC is characterized by a series of gene mutations, and the acquisition of the *KRAS* mutation is the earliest change in pancreatic carcinogenesis [10–12].

KRAS functions as a binary switch that cycles between an active GTP-bound and inactive GDP-bound state [13–16]. Mutant KRAS activates a diverse spectrum of downstream effector proteins [17], particularly components of the mitogen-activated protein kinase (MAPK) pathway and of the phosphoinositide-3-kinase (PI3K) pathway. *KRAS* mutation in PDAC is critical for reprogramming intracellular metabolism of, for example, glucose and glutamine, to facilitate the rapid proliferation of cancer cells [18–27]. To acquire sufficient nutrients for rapid proliferation, PDAC has elevated basal autophagy [28–30] in which biomolecules digested in lysosomes become available as nutrients [31, 32].

Although *KRAS* gene mutation is related to metabolic reprogramming in cancer cells [20, 26] or in genetically engineered mouse models [33, 34], it’s biological effects on metabolic reprogramming in human cells as early events in PDAC carcinogenesis are unclear. We report here dynamic metabolic reprogramming in immortalized human pancreatic duct epithelial cells with *KRAS* mutation (*KRAS*^G12V^) induced by gene-editing, which may mimic early pancreatic carcinogenesis. Although most studies of the *KRAS* oncogene involved overexpression of a mutant *KRAS* gene, these models do not always recapitulate early events of carcinogenesis because excessive expression of mutant KRAS driven by a constitutive promoter may affect its physiological function. The tissue of origin also determines whether transforming mutations initiate carcinogenesis [35]. Thus, we compared the phenotypes of heterozygous mutant *KRAS* genetically knocked-in to pancreatic epithelial cells and mutant *KRAS* gene-overexpressing cells, and mutant *KRAS* gene-overexpressing human colon epithelial cells.

## Results

### Establishment of human non-cancerous epithelial cells with *KRAS* mutation

**Fig. 1:**
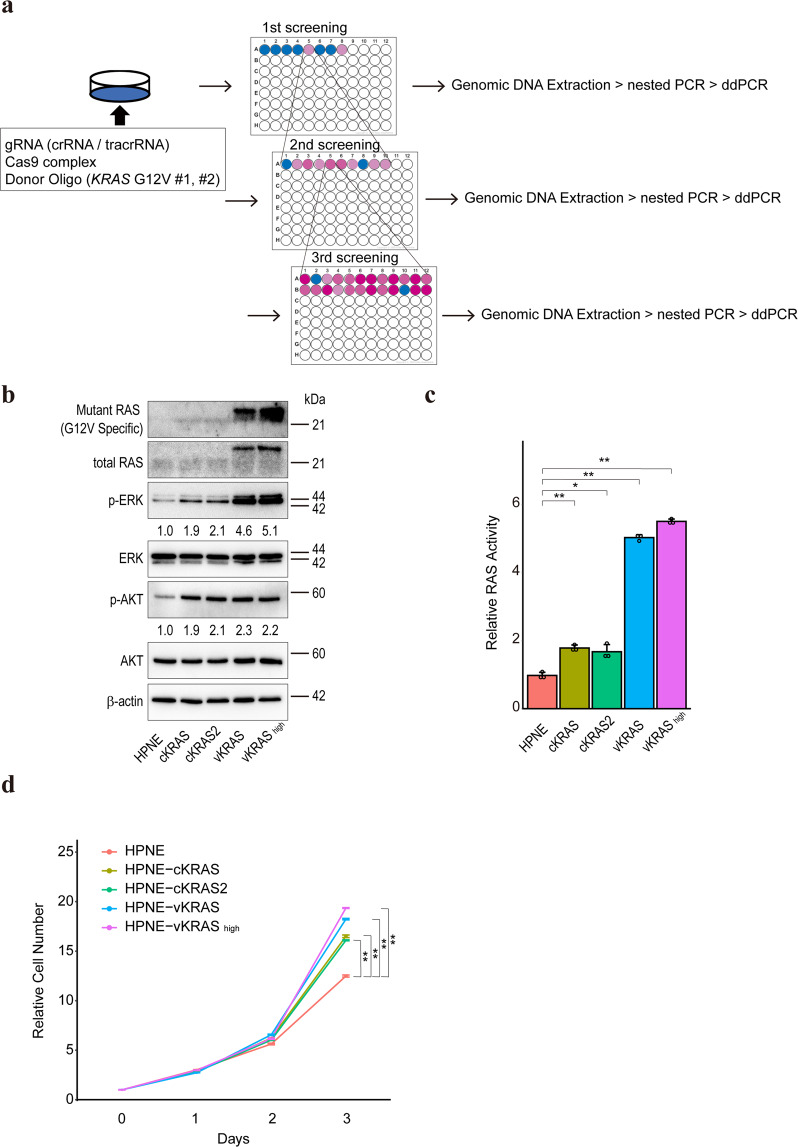
Establishment of gene-edited *KRAS*-mutant pancreatic epithelial cells. **a** Schema of cell selection. Gene-edited cells generated by transient transfection with gRNA, Cas9 complex, and ssODN, were sequentially selected by limiting dilution and ddPCR, yielding gene-edited monoclonal cells, blue and red wells, wild-type and *KRAS* mutant cells, respectively. **b** Determination of the levels of mutant KRAS (G12V) (genome-edited in cKRAS and HA-tagged mutant KRAS expression in vKRAS) and of related intracellular signaling molecules by Western blotting. The vKRAS construct has an HA-tag and is a slightly heavier molecule. Representative results from three independent experiments are shown. Band intensities of phosphorylated ERK (p-ERK) and phosphorylated AKT (p-AKT) are indicated below the images. **c** Determination of RAS activity by ELISA. Results are the average of three biological replicates; error bars represent the SD. *p < 0.05; **p < 0.01. **d** Cell growth curve. Cell numbers relative to those at day 0 are indicated. Results are the average of three biological replicates; error bars represent SD. **p < 0.01.

To investigate the biological effects of *KRAS* mutation in human pancreatic non-cancerous epithelial cells, we introduced using the clustered regularly interspaced short palindromic repeats (CRISPR)/Cas9 system in a G12V mutation in the *KRAS* gene in HPNE cells, an immortalized human pancreatic primary epithelial cell line. According to a previous report [36], to isolate cells with *KRAS* mutation repeated limiting dilution followed by assessment of mutation frequencies by droplet-digital PCR (ddPCR) was carried out (Fig. 1a). To eliminate the influence of the remaining ssODN, ddPCR was performed after nested PCR using primer pairs set outside the ssODN sequences. After four selection cycles, two clones, #152622 and #152623 were isolated, in which the *KRAS* gene mutation frequencies were 50%, indicating the successful introduction of heterozygous *KRAS* mutation (Supplementary Fig. 1a–d). Hereafter, we used clones #152622 (HPNE-cKRAS cells) and #152623 (HPNE-cKRAS2 cells). Lentivirus-mediated HA-tagged mutant KRAS (G12V) gene-overexpressing polyclonal HPNE cells (HPNE-vKRAS) and overexpressing immortalized non-cancerous human colon primary epithelial cells (colon-vKRAS), were used as references. To investigate the difference between HPNE-cKRAS and HPNE-vKRAS cells, we examined the mutant KRAS copy number in HPNE-vKRAS cells by subcloning several single clones. The *KRAS* gene mutation frequencies were unexpectedly nearly 33% in all isolated HPNE-vKRAS clones, indicating that only one copy of mutant KRAS was inserted into the genome, with two wildtype alleles (Supplementary Fig. 1e). However, mutant KRAS expression varied among clones; therefore, we examined the HPNE-vKRAS2 clone (hereafter HPNE-vKRAS_high_ cells) that expressed the highest amount of mutant KRAS in several experiments to examine whether biological functions were affected by the expression of mutant KRAS (Supplementary Fig. 1f).

Mutant KRAS expression levels in HPNE-cKRAS, HPNE-vKRAS, and colon-vKRAS cells were determined by western blotting using a KRAS G12V mutant-specific antibody (Fig. 1b and Supplementary Fig. 2a). Consistent with a previous report [37], phosphorylation of ERK and AKT was modestly induced in HPNE-cKRAS and HPNE-vKRAS cells (Fig. 1b). In contrast, the PI3K-AKT pathway was significantly activated, and ERK phosphorylation was modestly induced, in colon-vKRAS cells (Supplementary Fig. 2a). The RAS activities were almost in accordance with the levels of mutant KRAS protein expression (Fig. 1c and Supplementary Fig. 2b). The growth rate of HPNE-cKRAS and HPNE-vKRAS cells was increased (Fig. 1d), but that of colon-vKRAS cells was not (Supplementary Fig. 2c). Therefore, while *KRAS* mutation induces cell survival and intracellular signaling, the biological effects of *KRAS* mutation vary among tissues.

### The growth of *KRAS* mutant cells is dependent on glucose and glutamine

**Fig. 2:**
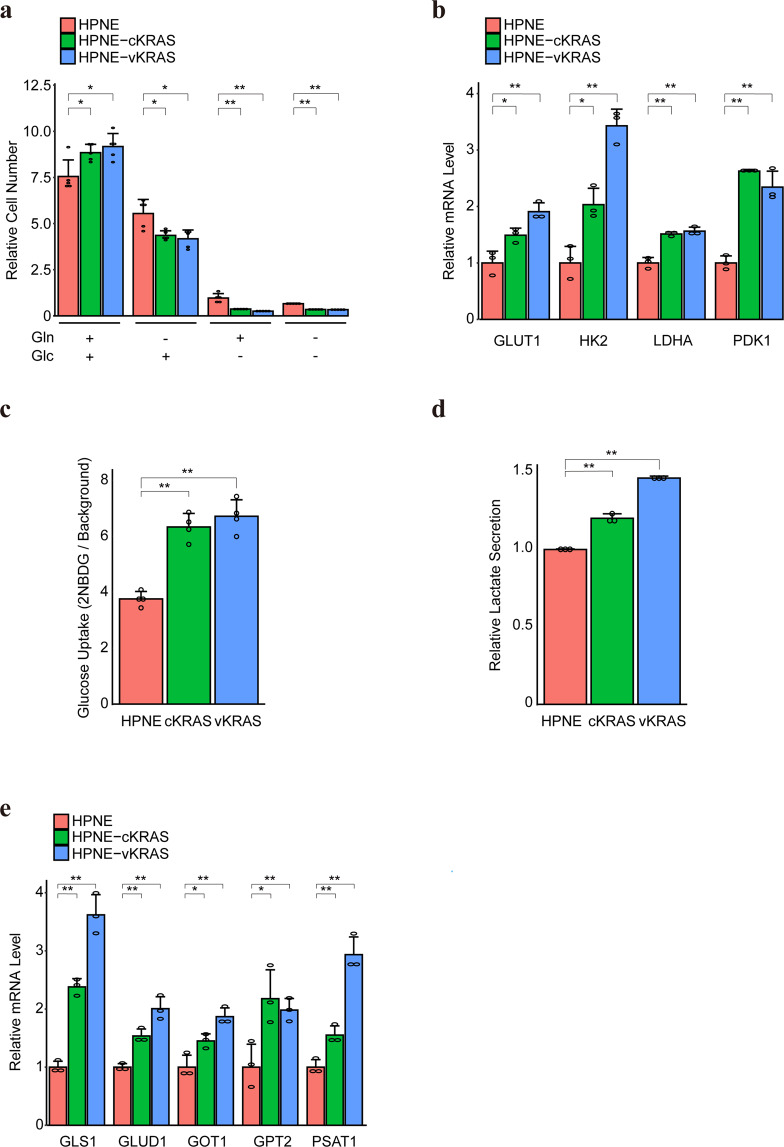
*KRAS* mutant pancreatic epithelial cells are dependent on glucose and glutamine. **a** Cell growth rate in the presence or absence of glucose (Glc) and glutamine (Gln). The relative cell number was determined by measuring cell numbers at days 0 and 3. Results are the means of five biological replicates. Error bars represent the SD. *p < 0.05; **p < 0.01. **b** Relative mRNA levels of the indicated genes. The levels in control HPNE cells were set as 1. Results are the means of three biological replicates; error bars represent the SD. *p < 0.05; **p < 0.01. **c** Glucose uptake levels were measured using 2-NBDG. Results are the average of four independent replicates. Error bars represent SD. **p < 0.01. **d** Lactate secretion levels are shown. Results are the average of three independent replicates. Error bars represent SD. **p < 0.01. **e** Relative mRNA levels of indicated genes are shown after setting the levels in HPNE cells as 1. Results are the average of three biological replicates. Error bars represent mean ± SD. *p < 0.05; **p < 0.01.

*KRAS* mutations alter glucose and glutamine metabolism in cancerous cells [18, 26]. To evaluate the metabolic changes induced by *KRAS* mutation in non-cancerous cells, we examined the dependence of glucose and/or glutamine on the growth of human non-cancerous pancreatic and colon epithelial cells with *KRAS* mutations. Compared to *KRAS* wild-type cells, the proliferation of HPNE-cKRAS and HPNE-vKRAS cells was significantly suppressed in glucose-deficient and/or glutamine-deficient medium (Fig. 2a). In colon epithelial cells, colon-vKRAS cells showed similar tendencies, although not significant regarding glutamine (Supplementary Fig. 3a). These results suggest that the growth of cells with *KRAS* mutation depends more on glucose or glutamine. Consistently, the expression levels of glucose transporter (GLUT1), rate-limiting glycolytic enzyme (HK2), and enzymes enhancing aerobic glycolysis (LDHA and PDK1) (Fig. 2b and Supplementary Fig. 3b–d), as well as glucose uptake (Fig. 2c and Supplementary Fig. 3e) were increased in HPNE-cKRAS, HPNE-vKRAS, and colon-vKRAS cells. In addition, the lactate level, which reflects enhanced glycolytic flux, was increased in HPNE-cKRAS and HPNE-vKRAS cells (Fig. 2d), whereas it was not significantly changed in colon-vKRAS cells (Supplementary Fig. 3f). Moreover, the expression levels of glutamine metabolism-related enzymes such as glutaminase 1 (GLS1), glutamate dehydrogenase 1 (GLUD1), and transaminases (GOT1, GPT2, and PSAT1) were increased (Fig. 2e and Supplementary Fig. 3c, d, and g), suggesting that glutamine utilization was increased in HPNE-cKRAS, HPNE-vKRAS, and colon-vKRAS cells. These different expression profiles of metabolic genes in mutant KRAS cells may be linked to the mutant KRAS expression and RAS activity levels (Fig. 1b and c).

Therefore, *KRAS* mutations modulate glucose and glutamine metabolism, which is required for the proliferation of non-cancerous cells with *KRAS* mutation.

### Glutamine is crucial for energy production and maintaining the redox balance in *KRAS* mutant cells

**Fig. 3:**
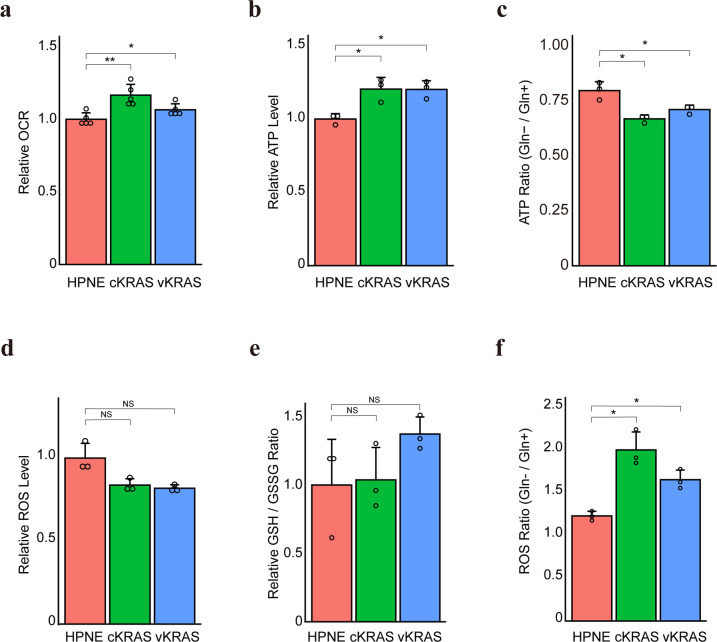
Glutamine is crucial for energy production and the redox balance in *KRAS* mutant pancreatic epithelial cells. **a** Oxygen consumption rate. Results are the means of five biological replicates; error bars represent the SD. *p < 0.05; **p < 0.01. **b** Intracellular ATP levels. Results are the means of three biological replicates; error bars represent the SD. *p < 0.05. **c** Intracellular ATP levels under normal and Gln-deficient conditions with the calculated ratios (ATP levels in Gln−/levels in Gln+). Results are the means of three biological replicates; error bars represent the SD. *p < 0.05. **d** Intracellular ROS levels. Results are the means of three biological replicates; error bars represent the SD. NS, not significant. **e** GSH/GSSG ratio assessed from the metabolome data and relative ratios taking the value of control HPNE cells as 1. Results are the means of three biological replicates; error bars represent the SD. NS, not significant. **f** Intracellular ROS levels under normal and Gln-deficient conditions with the calculated ratios (ROS levels in Gln−/levels in Gln+). Results are the means of three biological replicates; error bars represent the SD. *p < 0.05.

Glutamine is converted to α-ketoglutarate (α-KG), a substrate for the TCA cycle and energy production, nucleic acid synthesis, and amino acid synthesis [38]. Because mutant KRAS enhanced glutamine utilization, we hypothesized that the flow to the TCA cycle increased in non-cancerous cells with *KRAS* mutation, promoting mitochondrial oxidative phosphorylation, as in murine renal cells expressing oncogenic H-Ras [39]. As expected, the oxygen consumption rate (OCR), which reflects the level of mitochondrial oxidative phosphorylation (OXPHOS), was significantly increased in HPNE-cKRAS and HPNE-vKRAS cells (Fig. 3a). The mitochondrial DNA copy number, which is correlated with the expression levels of mitochondrial metabolic genes [40], was also significantly increased in HPNE-cKRAS and HPNE-vKRAS cells (Supplementary Fig. 4a). Subsequently, the ATP levels were increased in HPNE-cKRAS and HPNE-vKRAS cells, which reflects the enhanced glycolysis and OXPHOS (Fig. 3b). To examine the role of glutamine in energy production, *KRAS* mutant cells were cultured in a glutamine-deficient medium. The ATP levels were significantly decreased to a greater extent in HPNE-cKRAS and HPNE-vKRAS cells than in wild-type cells, compared with those cultured in glutamine-containing medium (Fig. 3c). Similarly, the ATP levels were significantly decreased in *KRAS* mutant cells following treatment with the glycolysis inhibitor 2-deoxy-d-glucose (2DG) and the OXPHOS inhibitor antimycin, which suggests that both the glycolysis and OXPHOS pathways are dependently utilized to maintain energy resources in the cells with the *KRAS* mutation (Supplementary Fig. 4b and c).

Intriguingly, although mitochondrial oxidative phosphorylation leads to the production of reactive oxygen species (ROS) [41], the ROS levels were decreased in HPNE-cKRAS and HPNE-vKRAS cells (Fig. 3d), suggesting that these cells maintained the redox balance despite enhanced mitochondrial metabolism. Consistently, the reduced glutathione (GSH)/oxidized glutathione (GSSG) ratio, an index of cellular redox metabolism [42], was maintained in those cells (Fig. 3e).

Glutamine is a substrate for the synthesis of glutathione, a tripeptide (composed of glutamate, cysteine, and glycine) that protects cells from free radical damage by acting as an antioxidant [38]. In PDAC with the *KRAS* mutation, glutamine metabolism is reprogramed to increase the NADPH/NADP^+^ ratio through the reactions of transaminases, which can help maintain the cellular redox state [26]. Therefore, based on the hypothesis that mutant KRAS increases glutathione synthesis via glutamine consumption to counter ROS production, *KRAS* mutant cells were cultured in a glutamine-deficient medium. The ROS levels were significantly increased to a greater extent in HPNE-cKRAS and HPNE-vKRAS cells than in wild-type cells, compared with those cultured in glutamine-containing medium (Fig. 3f). Consistently, the ROS levels were significantly increased in HPNE-cKRAS and HPNE-vKRAS cells compared with wild-type cells following treatment with the transaminase inhibitor aminooxyacetate (AOA) (Supplementary Fig. 4d). These findings suggest that non-cancerous pancreatic cells with *KRAS* mutation use glutamine for energy production, as well as maintaining the redox balance.

### Intracellular amino acid levels are decreased in *KRAS* mutant pancreatic epithelial cells

**Fig. 4:**
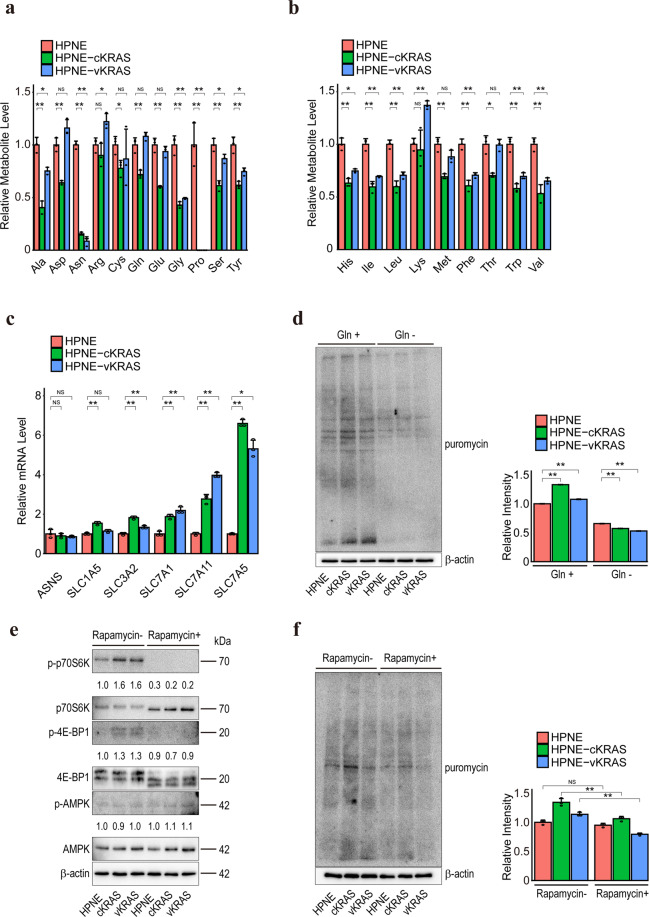
Intracellular amino acid levels are decreased in *KRAS* mutant pancreatic epithelial cells. **a** Intracellular non-essential amino acid levels. Results are the means of three biological replicates; error bars represent SD. NS, not significant; *p < 0.05; **p < 0.01. **b** Intracellular essential amino acid levels. Results are the means of three biological replicates; error bars represent SD. NS, not significant; *p < 0.05; **p < 0.01. **c** Relative mRNA levels of the indicated genes taking the levels in HPNE cells as 1. Results are the means of three biological replicates; error bars represent SD. NS, not significant; *p < 0.05; **p < 0.01. **d** Puromycin incorporation assay results. Cells were cultured in normal (Gln+) or glutamine-deficient (Gln−) medium for 24 h. Puromycin was added 10 min before sample collection and the lysates were subjected to Western blotting using an anti-puromycin antibody. Representative results from three independent experiments are shown. Relative total band intensities are shown at the right. Error bars represent SD. **p < 0.01. **e** mTOR pathway activity as determined by measuring the phosphorylation level of p70S6K and 4E-BP1 by Western blotting. Representative results from three independent experiments are shown. Band intensities are indicated below the images. **f** Puromycin incorporation assay results. Cells were treated with or without 100 nM rapamycin for 6 h. Puromycin was added 10 min before sample collection and the lysates were subjected to Western blotting using an anti-puromycin antibody. Representative results from three independent experiments are shown. Relative total band intensities are shown at the right. Error bars represent the SD. **p < 0.01. NS, not significant; **p < 0.01.

Because metabolic changes were observed in non-cancerous cells with *KRAS* mutation, we next performed comprehensive metabolome analyses of HPNE control, HPNE-cKRAS, and HPNE-vKRAS cells under normal culture conditions. Among the 116 metabolites examined, we focused on the amino acid levels. This was because, although almost all amino acids were reduced in HPNE-cKRAS and HPNE-vKRAS cells, non-essential amino acid levels, particularly asparagine and proline, were significantly reduced (Fig. 4a and b). Although the intracellular asparagine level was significantly reduced in HPNE-cKRAS and HPNE-vKRAS cells, the expression level of asparagine synthetase (ASNS) was not changed (Fig. 4c), suggesting that the reduction in the intracellular asparagine level was not a result of reduction of asparagine synthesis. The expression levels of most amino acid transporters were upregulated in HPNE-cKRAS, HPNE-vKRAS, and colon-vKRAS cells (Fig. 4c and Supplementary Fig. 5a). Based on these results, we speculate that amino acids, including asparagine, are transported from extracellular spaces and consumed in cells with *KRAS* mutation.

To evaluate the amino acid consumption and protein translation levels, we performed a puromycin labeling assay [43]. In a normal medium, protein synthesis was significantly enhanced in HPNE-cKRAS, HPNE-vKRAS, and colon-vKRAS cells (Fig. 4d and Supplementary Fig. 5b), particularly in HPNE-cKRAS cells (Fig. 4d). However, in a glutamine-deficient medium, protein synthesis was significantly and similarly suppressed irrespective of the *KRAS* mutation status (Fig. 4d and Supplementary Fig. 5b), probably because glutamine is required for uptake of essential amino acids [43, 44]. Consistently, mTOR activity, as indicated by the phosphorylated p70S6K and phosphorylated 4E-BP1 levels, was enhanced in HPNE-cKRAS and HPNE-vKRAS cells independent of AMPK status (Fig. 4e). Moreover, we observed that the mTOR inhibitor rapamycin suppressed protein synthesis (Fig. 4f). Collectively, these data suggest that the mTOR pathway is more activated and biomass synthesis is enhanced in cells with *KRAS* mutation, which leads to significant depletion of the intracellular amino acid levels.

### Autophagy is required for the maintenance of *KRAS* mutant cells

**Fig. 5:**
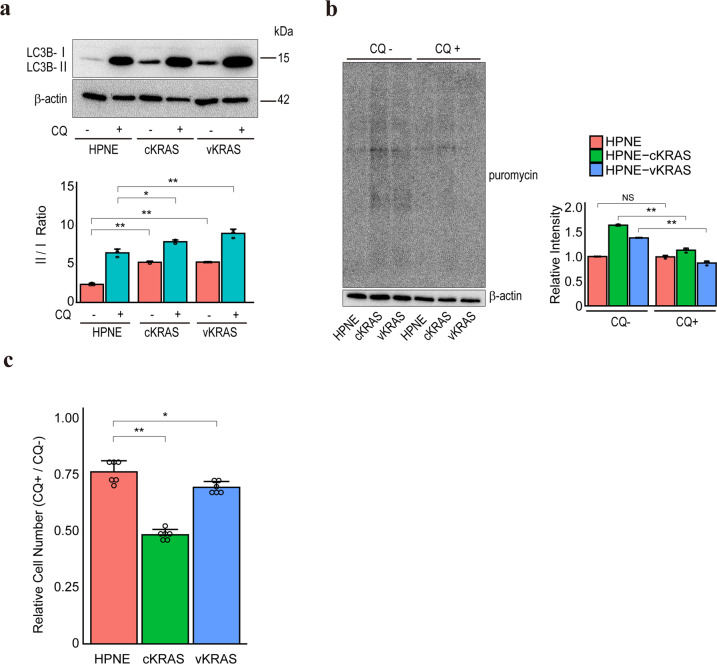
Autophagy is required for maintaining *KRAS* mutant pancreatic epithelial cells. **a** Autophagic flux levels as determined by Western blotting of LC3B-I and LC3B-II. Representative results from three independent experiments are shown. Band intensity ratios (LC3B-II/LC3B-I ratio) are indicated below the images. A lysosomal inhibitor, chloroquine (CQ), was used to assess autophagic flux by assessing the LC3B-II level. Error bars represent the SD. *p < 0.05; **p < 0.01. **b** Puromycin incorporation assay results. Cells were treated with or without 5 μM chloroquine for 4 h. Puromycin was added 10 min before sample collection. Representative results from three independent experiments are shown. Relative total band intensities are shown at the right. Error bars represent the SD. NS, not significant; **p < 0.01. **c** Cells were treated with 5 μM chloroquine for 48 h. The number of cells relative to the control (without treatment) was determined. Results are the means of six biological replicates; error bars represent the SD. *p < 0.05; **p < 0.01.

Amino acids, such as asparagine and glutamine, are essential for cell growth [43–45]. Because amino acid consumption was enhanced in HPNE-cKRAS and HPNE-vKRAS cells, rendering them nutrient-deficient, we hypothesized that autophagy is upregulated to promote amino acid-recycling. As expected, the autophagic flux was increased in HPNE-cKRAS, HPNE-vKRAS, and colon-vKRAS cells compared to control cells (Fig. 5a and Supplementary Fig. 6a). Accordingly, the protein synthesis in HPNE-cKRAS and HPNE-vKRAS cells (Fig. 5b) and the proliferation of HPNE-cKRAS, HPNE-vKRAS, and colon-vKRAS cells (Fig. 5c and Supplementary Fig. 6b) were significantly suppressed by the autophagy inhibitor chloroquine, suggesting that autophagy provides essential nutrient resources for the viability of *KRAS* mutant cells. To investigate further mechanisms of autophagy activation in *KRAS* mutant cells, we evaluated the autophagic flux with MEK inhibitor treatment. Consistent with previous reports with PDAC cells [46, 47], the inhibition of MEK, a downstream molecule of KRAS, activates autophagic flux regardless of KRAS state (Supplementary Fig. 7), which indicated that other downstream pathways could regulate autophagic flux in HPNE-cKRAS and HPNE-vKRAS cells.

These findings imply that nutritional interventions with autophagy inhibition in non-cancerous epithelial cells can suppress or delay the development of PDAC.

## Discussion

*KRAS* mutation increased the dependency on glucose and glutamine for energy production and maintenance of the redox balance in human non-cancerous pancreatic cells. In addition, the intracellular amino acid levels were significantly decreased. Accordingly, the cells required greater autophagic flux for proliferation, as did tumor maintenance in PDAC mouse models [30, 48, 49]. Therefore, nutritional interventions and autophagy inhibition may prevent the proliferation of non-cancerous pancreatic cells with *KRAS* mutation.

Evidence of the existence of metabolic reprogramming in *KRAS*-mutated PDAC cells has accumulated [18–23]. Our results also indicated that pancreatic non-cancerous cells with induced *KRAS* mutation are dependent on glucose and glutamine. Because pancreatic carcinogenesis is accompanied by *KRAS* mutation as an initial molecular event [10–12], these results suggest that metabolic reprogramming is induced by *KRAS* mutation at an early stage of pancreatic carcinogenesis.

Non-cancerous pancreatic cells with *KRAS* mutation exhibited markedly decreased intracellular amino acid levels. The mTOR pathway was upregulated in cells with *KRAS* mutation, indicating enhanced protein synthesis and amino acid consumption [44]. In addition, several amino acid transporters were highly expressed to meet the increased demand for amino acids in cells with *KRAS* mutation, as reported in PDAC [50, 51]. Among amino acids, asparagine and proline were markedly decreased in non-cancerous pancreatic cells with *KRAS* mutation. Because intracellular asparagine is exchanged for extracellular amino acids to regulate protein synthesis [52], asparagine may be consumed not only for protein synthesis but also to enhance the importation of other amino acids. In addition, because PDAC cells maintain their growth by producing proline as a substrate for the TCA cycle [53], the reduced proline levels in pancreatic cells with *KRAS* mutation are likely to reflect increased consumption of proline in the TCA cycle. Therefore, pancreatic cells with *KRAS* mutation may have extensive reprogramming of amino acid metabolism to maintain their viability.

Autophagic flux was increased in cells with *KRAS* mutation in this study, consistent with studies of PDAC [28, 48]. However, it remains to be clarified how KRAS drives the high basal levels of autophagy. To identify the relationship between *KRAS* mutation, KRAS activation, and autophagy, we examined the autophagic flux after treatment with an inhibitor of MEK, which is downstream of KRAS. MEK inhibition elevated rather than decreased the autophagic flux, as reported in PDAC [46, 47]. Although this may be a consequence of disrupted glycolysis and mitochondrial function by MEK inhibition, as discussed in PDAC, further study is needed to identify the direct and indirect regulators that lead to the compensatory increased autophagic flux induced by *KRAS* mutation.

*KRAS* mutation is crucial for carcinogenesis in various tissues, not only the pancreas. Human colon polyclonal epithelial cells with *KRAS* mutation exhibited increased autophagic flux and were sensitive to autophagy inhibition, similar to pancreatic polyclonal epithelial cells with *KRAS* mutation. However, their phenotype was slightly different from those of pancreatic cells, in terms of, for example, PI3K pathway activity and ASNS expression, as reported previously [54]. Therefore, the *KRAS* mutation is universally crucial for carcinogenesis, its biological effects may be tissue or cell-type specific, as reported [55].

Gene-editing technology enables the examination of the effects of mutations. We compared the physiological effect of *KRAS* mutation using mutant knocked-in HPNE cells and *KRAS* gene-overexpressing polyclonal cells. Although the phenotypes were mostly similar, several quantitative assays, such as protein translation levels and sensitivities to autophagy inhibition, gave different results. This is likely to be caused by differences in the mutant KRAS expression levels. Indeed, the mutant KRAS allelic content reportedly defines the degree of metabolic reprogramming [56] and *KRAS* gene amplification occurs during pancreatic carcinogenesis [57]. Therefore, mutation knock-in cells are more physiological and suitable for investigations of the early steps of carcinogenesis. Further, the effects of the lentiviral backbone used to generate the mutant *KRAS* gene-overexpressing cells cannot be ignored. Therefore, caution is needed when interpreting the results and performing functional analysis of mutant gene-overexpressing cells.

In conclusion, *KRAS* mutation in human pancreatic ductal epithelial cells triggered diverse metabolic reprogramming and compensatory autophagy, which may create a favorable environment for cell proliferation and accelerate the progression to carcinogenesis. Although the pancreatic epithelial cells used in this study were immortalized, the results may provide us with the biological effects in the early phase of carcinogenesis, initialized by *KRAS* mutation in humans. Because the existence of cells with *KRAS* mutation in the body has recently been detected from plasma using cell-free DNA with high sensitivity [58], nutritional intervention including autophagy inhibition from the early stages of carcinogenesis may become an option to suppress or delay subsequent carcinogenesis in the future.

## Methods

### Cell lines

Human normal pancreatic epithelial (HPNE) cells immortalized with hTERT and human pancreatic carcinoma (CFPAC-1) cells carrying heterozygous *KRAS*^G12V^ mutation with KRAS copy number gain [59, 60] were obtained from the American Type Culture Collection (Manassas, VA). 293TN human embryonic kidney cells were purchased from System Biosciences (SBI, Mountain View, CA). Human normal colonic epithelial cells immortalized with SV40 large T antigen were purchased from Applied Biological Materials (Applied Biological Materials, Richmond, BC, Canada). HPNE cells and 293TN cells were cultured in Dulbecco’s Modified Eagle’s Medium (DMEM) (Sigma-Aldrich, St. Louis, MO, #D6046) supplemented with 10% fetal bovine serum (FBS). CFPAC-1 cells were cultured in Iscove’s Modified Dulbecco’s Medium (IMDM) (Sigma-Aldrich, #I3390) supplemented with 10% FBS. Human colonic epithelial cells were cultured in DMEM supplemented with 5% FBS. All cells were incubated at 37 °C in an atmosphere containing 20% O_2_ and 5% CO_2_.

### Nutrient-deficient media

Glucose-free and glutamine-free DMEM was purchased from Sigma-Aldrich (#D5030) and supplemented with sodium bicarbonate (Wako Pure Chemical Industries, Osaka, Japan) and FBS. Immediately before use, glucose (Wako Pure Chemical Industries) and/or glutamine (Invitrogen, Carlsbad, CA) were added to 5.5 and 4 mM, respectively. For puromycin labeling assay and cellular ROS measurement, glutamine-free DMEM (Sigma-Aldrich, #D5546) with FBS was used.

### Genome editing using the CRISPR/Cas9 system

Cas9 protein, CRISPR RNA (crRNA) (*KRAS* #1 and *KRAS* #2), and trans-crRNA (tracrRNA) were purchased from Integrated DNA Technologies (IDT, Coralville, IA). The crRNAs used were as follows: *KRAS* #1, 5’-rCrUrGrArArUrUrArGrCrUrGrUrArUrCrGrUrCrArGrUrUrUrUrArGrArGrCrUrArUrGrCrU-3’ and *KRAS* #2, 5’-rGrArArUrArUrArArArCrUrUrGrUrGrGrUrArGrUrGrUrUrUrUrArGrArGrCrUrArUrGrCrU-3’. The single-stranded oligo DNAs (ssODNs) were purchased from Eurofins Genomics (Tokyo, Japan). The ssODNs used were as follows: ssODN for *KRAS* #1, 5’-TTTTCATTATTTTTAATTTTGTGGACGAAT-3’; ssODN for *KRAS* #2, 5’-TACCTCTATTGTTGGATTTTCAGCAGGCCT-3’. HPNE cells were transfected according to the recommended protocol of IDT. To isolate cells with *KRAS* mutation, the mutation frequencies were examined by droplet-digital PCR (ddPCR) after repeated limiting dilution of the samples. Briefly, single-cell clones were seeded in a 96-well plate, cultured for several days, and genomic DNA was extracted from a randomly selected well using a DNA Extraction Kit (Kaneka, Tokyo, Japan). After nested PCR using primer pairs outside the ssODN sequences, ddPCR was performed using probes (Bio-Rad Laboratories Hercules, CA) for simultaneous detection of *KRAS* mutant allele (FAM)/wild allele (HEX) (#10049550). CFPAC-1 cells were used as the positive control and HPNE cells as the negative control. Cell populations with high mutant frequencies were selected and seeded in 96-well plates with limiting dilution. The above procedure was repeated four times to concentrate the mutant allele to isolate cells heterozygous for a *KRAS* mutant allele. The primer sequences used are listed in Supplementary Table 1.

### Transfection and lentivirus transduction

For lentivirus-mediated KRAS^G12V^ overexpression, pLenti-PGK-KRAS4B (G12V) was obtained from Addgene (plasmid #35633). The Lentivirus Packaging System (SBI) was used to generate stably expressing polyclonal cells according to the manufacturer’s protocol. Briefly, the pLenti-PGK-KRAS4B (G12V) plasmid and pPACKH1 packaging plasmid mix were transfected into 293TN cells using Effectene Transfection Reagent (QIAGEN, Hilden, Germany). After 48 h, the culture media were collected and the viruses were concentrated using PEG-it Reagent (SBI). The centrifuged pellet was resuspended in 1× PBS, and aliquots were stored at −80 °C. Viruses were added to the target cells with polybrene reagent (Santa Cruz Biotechnology, Dallas, TX), followed by selection with hygromycin.

### Cell growth assay

The Cell Counting Kit-8 (Dojindo Molecular Technologies, Kumamoto, Japan) was used to assay cell growth according to the manufacturer’s protocol. Briefly, 1.0 × 10^4^ cells were seeded in 96-well plates. Next, 10 µL of CCK-8 solution were added, the plates were incubated for 60 min at 37 °C, and the absorbance at 450 nm was measured using a microplate reader (Thermo Fisher Scientific, Waltham, MA).

### RAS activity assay

RAS activity was measured using the 96-well Ras Activation ELISA Kit (Cell Biolabs, San Diego, CA). Briefly, 4.0 × 10^5^ cells were cultured on a 6-well dish and lysate samples were extracted using Assay/Lysis Buffer. Then, the lysates were added to each well of the plate and the active form of RAS in the samples was selectively isolated with plate-bound Raf-1 RBD, followed by incubation with an anti-pan-Ras antibody and HRP-conjugated secondary antibody. The absorbance was measured at 450 nm by a microplate reader (Thermo Fisher Scientific).

### Western blotting and antibodies

Western blotting was performed as described previously [61]. Briefly, lysate samples were separated in 10–20% gradient polyacrylamide gels and transferred to polyvinylidene difluoride membranes (GE Healthcare, Little Chalfont, UK). After blocking with 5% dry milk, the membranes were probed with the appropriate primary antibodies diluted in Immunoshot Reagent 1 (Cosmo bio, Tokyo, Japan). The corresponding horseradish peroxidase (HRP)-conjugated secondary antibodies (GE Healthcare) were subsequently added. Bound antibodies were detected using the Immunostar LD Reagents (Wako Pure Chemical Industries). Antibodies against the following were used: β-actin (#5125, 1:10000), Ras (G12V Mutant Specific) (D2H12) (#14412, 1:1000), Ras (#3965, 1:1000), phospho-p44/42 MAPK (Erk1/2) (Thr202/Tyr204) (#9101, 1:1000), p44/42 MAPK (Erk1/2) (#9102, 1:1000), phospho-Akt (Ser473) (D9E) (#4060, 1:1000), Akt (#9272, 1:1000), Hexokinase II (C64G5) (#2867, 1:1000), LDHA (C4B5) (#3582, 1:1000), phospho-p70 S6 kinase (Thr389) (#9205, 1:1000), p70 S6 kinase (49D7) (#2708, 1:1000), phospho-4E-BP1 (Thr70) (#9455, 1:1000), 4E-BP1 (53H11) (#9644, 1:1000), phospho-AMPKα (Thr172) (D4D6D) (#50081, 1:1000), AMPKα (D5A2) (#5831, 1:1000), and LC3B (D11) (#3868, 1:1000) (Cell Signaling Technology, Danvers, MA). GLUD1 (ab166618, 1:1000) (Abcam, Cambridge, MA). GOT1 (AV48205, 1:1000) (Sigma). Uncropped images are in Supplementary Figs. 8 and 9.

### Puromycin-labeling assay

The puromycin-labeling assay was performed as described previously [43]. Briefly, cells were cultured in the presence and absence of glutamine for 24 h. Puromycin (90 mM) was added to the culture medium for 10 min, and the cells were rigorously washed and harvested for Western blotting using an anti-puromycin antibody (MABE343, 1:1000; Merck Millipore, Burlington, MA).

### RNA extraction, reverse transcription, and quantitative real-time PCR

Total RNA was isolated from 1.0 × 10^6^ cells using ISOGEN II (Nippon Gene, Toyama, Japan) according to the manufacturer’s protocol. Reverse transcription was performed using the SuperScript III First-Strand Synthesis System for RT-PCR (Invitrogen). Next, 100 ng of template were subjected to quantitative SYBR Green PCR (StepOnePlus™ Realtime PCR System; Invitrogen). All qPCR tests were run in triplicate. The average threshold cycle number (Ct) values were normalized to that of actin and calculated using the delta-delta Ct (ddCt) method. The primer sequences used are listed in Supplementary Table 2.

### Glucose-uptake assay

Glucose-uptake assay was performed as described previously [62]. Briefly, 4.0 × 10^5^ cells were treated with 100 μΜ fluorescently labeled deoxyglucose analog, 2-(N-(7-nitrobenz-2-oxa-1,3-diazol-4-yl)amino)-2-deoxyglucose (2-NBDG; Invitrogen) [63] for 30 min. The cells were trypsinized and single-cell suspensions were analyzed by flow cytometry using Guava Easy Cyte Plus (GE Healthcare). As a result of differences in endogenous fluorescence between cells, 2NBDG-mediated fluorescence intensities were normalized to those of unlabeled cells.

### Cellular lactate measurement

The cellular lactate level was measured using the Lactate Assay Kit-WST (Dojindo Molecular Technologies) according to the manufacturer’s protocol. Briefly, 1.0 × 10^4^ cells were seeded in 96-well plates, and the plates were incubated overnight at 37 °C. The medium was exchanged for fresh medium, and the cells were incubated for 24 h. Next, 20 μL of culture supernatant was transferred to a 96-well plate, 80 μL of the working solution was added, and the plates were incubated for 30 min at 37 °C. Finally, the absorbance at 450 nm was measured using a microplate reader (Thermo Fisher Scientific).

### Measurement of oxygen consumption rate

Oxygen consumption rate was measured using an Oxygen Consumption Rate Assay Kit (Cayman Chemical, Ann Arbor, MI) according to the manufacturer’s protocol. Briefly, 2.0 × 10^4^ pre-cultured cells in 96-well plates were incubated overnight at 37 °C. The medium was then replaced with 160 µL of fresh medium, and a phosphorescent probe was added to measure the oxygen consumption. Each well was sealed with 100 µL of mineral oil to prevent oxygen diffusion. The signals were measured by an ARVO X5 plate reader (PerkinElmer Japan, Kanagawa, Japan) using time-resolved mode at Ex 380 nm and Em 650 nm for 210 min at 1-min intervals. Linear regression was performed after subtracting the blank, and the oxygen consumption rate was indicated by the slope of each signal profile.

### Assessment of mitochondrial DNA copy number

Total DNA was isolated from 1.0 × 10^6^ cells using the QIAamp DNA Mini Kit (QIAGEN) according to the manufacturer’s protocol. Next, 100 ng of template were subjected to qPCR. The average Ct values of mitochondrial and nuclear DNA were obtained. The relative content of mitochondrial DNA, normalized to that of nuclear DNA, was calculated using the ddCt method. As primers, Human Mitochondrial DNA (mtDNA) Monitoring Primer Sets (TaKaRa, Shiga, Japan) targeting ND1 and ND5 as mitochondrial DNAs and SLCO2B1 and SERPINA1 as nuclear DNAs were used.

### Assay of cellular ATP levels

Cellular ATP levels were measured using the CellTiter-Glo Luminescent Cell Viability Assay Kit (Promega, Fitchburg, WI). Briefly, 1.0 × 10^4^ cells were cultured in 100 µL of the medium on 96-well plates. Next, 100 µL of CellTiter-Glo Reagent was added and mixed for 2 min on an orbital shaker to induce cell lysis. Subsequently, the plates were incubated for 10 min at RT, and the luminescence was measured using a GloMax 96 Microplate Luminometer (Promega).

### Assay of cellular ROS levels

Cellular ROS levels were measured using the ROS-Glo H_2_O_2_ Assay Kit (Promega). Briefly, 1.0 × 10^4^ cells were cultured in 80 µL of the medium on 96-well plates. Next, 20 µL of the H_2_O_2_ substrate solution was added, followed by incubation for 6 h in the indicated medium. Subsequently, 100 µL of ROS-Glo Detection Solution were added and the plates were incubated for 20 min at RT, followed by measurement of luminescence using a GloMax 96 Microplate Luminometer (Promega).

### Metabolome analysis

Metabolome analysis was performed by Human Metabolome Technologies (HMT, Tsuruoka, Japan), according to their methods. Briefly, 1.0 × 10^6^ cells were seeded onto 10 cm dishes for 24 h, and metabolites were extracted. The cells were washed twice with 5% mannitol and treated with 800 mL of methanol and 550 mL of water containing internal standards (HMT). The extract was filtered using an UltrafreeMC-PLHCC 5 kDa-cutoff filter unit (HMT) by centrifugation at 9100×*g*, at 4 °C for 120 min. Metabolite extracts were prepared as biological triplicates for each sample. Cationic compounds were measured in the positive mode of CE-TOFMS, and anionic compounds were measured in the positive and negative modes of CE-MS/MS as described previously [64]. The peaks detected by CE-TOFMS and CE-MS/MS were extracted using automatic integration software [65] (MasterHands, Keio University, Tsuruoka, Japan) and MassHunter Quantitative Analysis B.04.00, Agilent Technologies, Santa Clara, CA) to obtain peak information, including *m*/*z*, migration time (MT), and peak area. The peaks were annotated with putative metabolites from the HMT metabolite database based on their MTs in CE and *m*/*z* values determined by TOFMS and MS/MS. The tolerance range for the peak annotation was set to ±0.5 min for MT and ±10 ppm for *m*/*z*. Metabolite concentrations were calculated by normalizing the peak area of each metabolite to that of the internal standard and by using standard curves generated from three-point calibrations.

### Analysis of autophagic flux

Cells were treated with chloroquine (5 μM) for 2 h. Autophagic flux was quantified by determining the ratio of the band intensities of LC3II to LC3BI after Western blotting.

### Statistical analysis

The statistical significance of differences between groups was evaluated by Student’s *t*-test when the variances were equal. When the variances were unequal, Welch’s *t*-test was used instead. *P*-values less than 0.05 were considered to indicate statistical significance.

## Supplementary information

Supplementary Information

## Data Availability

The authors declare that all the data supporting the findings of this study are available within the article and its supplementary information files and from the corresponding author upon reasonable request.
