# Supplementary material for: Mutant KRAS drives metabolic reprogramming and autophagic flux in premalignant pancreatic cells
Source: Cancer Gene Ther. 2021 Apr 8;29(5):505–18. doi: 10.1038/s41417-021-00326-4 (PMC9113932; doi:10.1038/s41417-021-00326-4)

Supplementary Information

Mutant KRAS drives metabolic reprogramming and autophagic flux in premalignant pancreatic cells

Tatsunori Suzuki, Takahiro Kishikawa, Tatsuyuki Sato, Norihiko Takeda, Yuki Sugiura, Takahiro Seimiya, Kazuma Sekiba, Motoko Ohno, Takuma Iwata, Rei Ishibashi, Motoyuki Otsuka, and Kazuhiko Koike

Inventory of Supplementary Information:

Supplementary Figure Legends
Supplementary Table 1-2
Supplementary Figures 1-9

SUPPLEMENTARY FIGURE LEGENDS

Supplementary Figure 1. Establishment of gene-edited *KRAS*-mutant HPNE cells.

a, HPNE cells transfected with ssODN (*KRAS*#1) were seeded in 96-well plates, seven wells of which were randomly extracted and designated #11–#17. Similarly, those derived from ssODN (*KRAS* #2) were designated #21–#27. As shown in Figure 1a, ddPCR was performed. The cell population with a high mutation rate (#15) was selected and used for further selection by limited dilution followed by the determination of the mutation ratio by ddPCR. **b**, **c**, **d**, #152 in (b) and #1526 in (c) were subjected to further selection. Finally, #152622 and #152623 in (d) were selected as clones with induced *KRAS* mutations, possibly heterozygotes, with an approximately 50% mutation rate. HPNE cells without transfection were used as a negative control (NC) and CFPAC-1 cells, heterozygous *KRAS* mutant pancreatic cancer cells with copy number variation, were used as a reference. **e**, Determination of mutant KRAS copy number after subcloning HPNE-vKRAS cells (HPNE-vKRAS1–9). **f**, Determination of mutant KRAS expression levels by Western blotting. HPNE-vKRAS2 was selected as the clone that expressed the highest level of mutant KRAS. Representative results from three independent experiments are shown.

Supplementary Figure 2. Establishment of mutant *KRAS*-expressing human colon epithelial cells.

a, Determination of mutant KRAS (G12V) expression and the levels of related intracellular signaling molecules in colon-vKRAS cells by Western blotting. Representative results from three independent experiments are shown. Band intensities of phosphorylated ERK (p-ERK) and phosphorylated AKT (p-AKT) are indicated below the images. **b,** Determination of RAS activity in colon cells and colon-vKRAS cells by ELISA. Results are the average of three biological replicates; error bars represent the SD. $**p < 0.01$. **c,** Cell growth curve. Cell

numbers relative to those at day 0 are indicated. Results are the average of three biological replicates; error bars represent SD. NS, not significant.

Supplementary Figure 3. Mutant *KRAS*-expressing cells are dependent on glucose and glutamine.

a, Cell growth rate in the presence or absence of glucose (Glc) and glutamine (Gln). Relative cell number was determined as the ratio of that at day 0 and day 3. Results are the average of three biological replicates; error bars represent SD. NS, not significant; $*p < 0.05$; $**p < 0.01$. **b,** Relative mRNA levels of the indicated genes. The levels in control colon epithelial cells were set as 1. Results are the means of three biological replicates; error bars represent SD. $*p < 0.05$; $**p < 0.01$. **c, d,** Determination of the levels of enzymes related to glycolysis and glutamine metabolism in pancreatic (c) and colon (d) cells by Western blotting. Representative results from three independent experiments are shown. **e,** Glucose uptake measured using 2-NBDG. Results are the means of five independent replicates; error bars represent SD. $*p < 0.05$. **f,** Lactate levels. Results are the means of three independent replicates; error bars represent SD. NS, not significant. **g,** Relative mRNA levels of the indicated genes after setting the levels in control colon cells as 1. Results are the means of three biological replicates; error bars represent SD. NS, not significant; $**p < 0.01$.

Supplementary Figure 4. Glutamine is crucial for energy production and the redox balance in *KRAS* mutant pancreatic epithelial cells.

a, Mitochondrial DNA (mtDNA) copy number, as measured by qPCR. Results are the means of three biological replicates; error bars represent the SD. $*p < 0.05$. **b**, Cells were treated with or without 10 mM 2DG for 10 min. Intracellular ATP levels relative to the controls

(without treatment) were measured. Results are the means of three biological replicates; error bars represent the SD. *$p < 0.05$. **c**, Cells were treated with or without 10 μM antimycin for 10 min. Intracellular ATP levels relative to the controls (without treatment) were measured. Results are the means of three biological replicates; error bars represent the SD. *$p < 0.05$; **$p < 0.01$. **d**, Cells were treated with or without 2 mM AOA for 3 h. Intracellular ROS levels relative to the controls (without treatment) were measured. Results are the means of three biological replicates; error bars represent the SD. *$p < 0.05$.

Supplementary Figure 5. Protein synthesis is significantly increased in mutant KRAS-expressing colon epithelial cells.

a, Relative mRNA levels of the indicated genes in colon-vKRAS cells; the levels in control colon epithelial cells were set as 1. Results are the means of three biological replicates; error bars represent the SD. *$p < 0.05$; **$p < 0.01$. **b**, Puromycin incorporation assay results. Cells were cultured in normal (Gln+) or glutamine-deficient (Gln−) medium for 24 h. Puromycin was added 10 min before sample collection and the lysates were subjected to Western blotting using an anti-puromycin antibody. Representative results from three independent experiments are shown. Relative band intensities are shown at right. Error bars represent the SD. NS, not significant; **$p < 0.01$.

Supplementary Figure 6. Autophagy is required for maintaining mutant KRAS-expressing colonic epithelial cells.

a, Autophagic flux was assayed by Western blotting of LC3B-I and -II in colon-vKRAS cells. Representative results from three independent experiments are shown. Band intensity ratios (LC3B-II/LC3B-I ratio) are indicated below the images. A lysosomal inhibitor, chloroquine (CQ), was used to assess autophagic flux. Error bars represent the SD.

$p < 0.01$. **b, Chloroquine effects on cell proliferation. Cells were treated with 5 μM chloroquine for 48 h. The cell numbers relative to the controls (without treatment) were measured. Results are the means of six biological replicates. Error bars represent the SD. *$p < 0.05$.

Supplementary Figure 7. MEK inhibition increases autophagic flux.

Cells were treated with or without 10 μM selumetinib, a MEK inhibitor, for 8 h. Autophagic flux levels as determined by Western blotting of LC3B-I and -II. Representative results from three independent experiments are shown. Band intensity ratios (LC3B-II/LC3B-I ratio) are indicated below the images.

Supplementary Figure 8. Full blot images for the main figures.

Full-length Western blotting for Figures 1b, 4d, 4e, 4f, 5a, and 5b.

Supplementary Figure 9. Full blot images for the supplementary figures.

Full-length Western blotting for Supplementary Figures 1f, 2a, 3c, 3d, 5b, 6a, and 7.

Gene		Sequence (5'→3')
KRAS	Fw	GTATTAACCTTATGTGTGACATG
	Rv	GGTCCTGCACCAGTAATATGC

Supplementary Table 1. Primers used for nested PCR.

Gene		Sequence (5'→3')
GLUT1	Fw	GATTGGCTCCTTCTCTGTGG
	Rv	TCAAAGGACTTGCCCAGTTT
HK2	Fw	GAGCCACCACTCACCCTACT
	Rv	ACCCAAAGCACACACGGAAGTT
LDHA	Fw	TTGACCTACGTGGCTTGGAAG
	Rv	GGTAACGGAATCGGGCTGAAT
PDK1	Fw	CTGTGATACGGATCAGAAACCG
	Rv	TCCACCAAACAATAAAGAGTGCT
GLS1	F	TAGTTGTCCCCAATGTTATGGGT
	R	AGTCCAATGGTCCAAAGGAATG
GLUD1	Fw	GGGATTCTAACTACCACTTGCTCA
	Rv	AACTCTGCCGTGGGTACAAT
GOT1	Fw	CAACTGGGATTGACCCAACT
	Rv	GGAACAGAAACCGGTGCTT
GPT2	Fw	CATGGACATTGTCGTGAACC
	Rv	TTACCCAGGACCGACTCCTT
PSAT1	Fw	CGGTCCTGGAATACAAGGTG
	Rv	AACCAAGCCCATGACGTAGA
ASNS	Fw	GGAAGACAGCCCCGATTTACT
	Rv	AGCACGAACTGTTGTAATGTCA
SLC1A5	Fw	TCATGTGGTACGCCCCTGT
	Rv	GCGGGCAAAGAGTAAACCCA
SLC3A2	Fw	CTGGTGCCGTGGTCATAATC
	Rv	GCTCAGGTAATCGAGACGCC
SLC7A1	Fw	GCCTGTGCTATGGCGAGTTT
	Rv	ACGCTTGAAGTACCGATGATGTA
SLC7A11	Fw	GCGTGGGCATGTCTCTGAC
	Rv	GCTGGTAATGGACCAAAGACTTC
SLC7A5	Fw	CCGTGAACTGCTACAGCGT
	Rv	CTTCCCGATCTGGACGAAGC
Actin	Fw	CATGTACGTTGCTATCCAGGC
	Rv	CTCCTTAATGTCACGCACGAT

Supplementary Table 2. Primers used for RT-qPCR.

Supplementary Figure 1

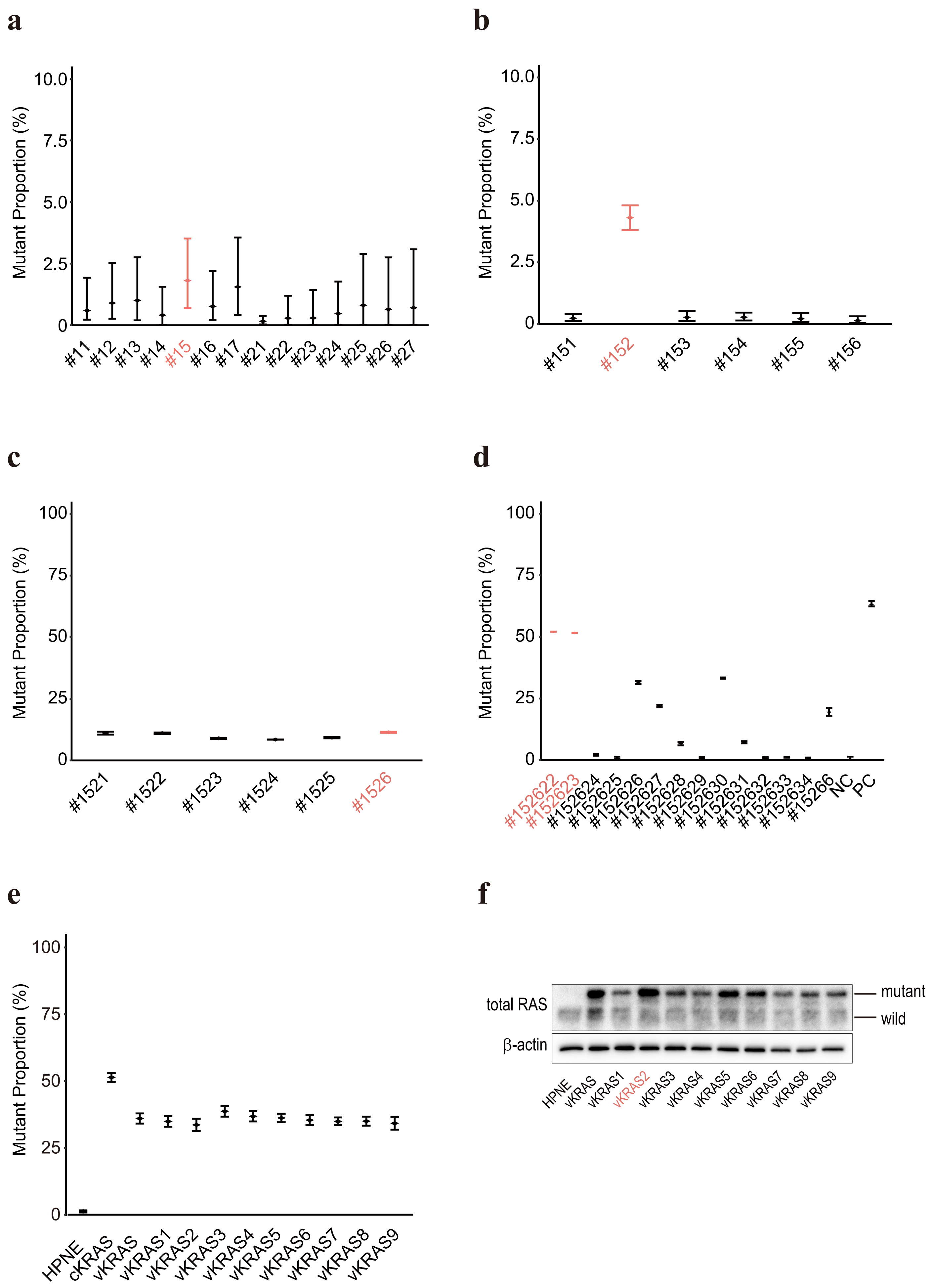

Supplementary Figure 2

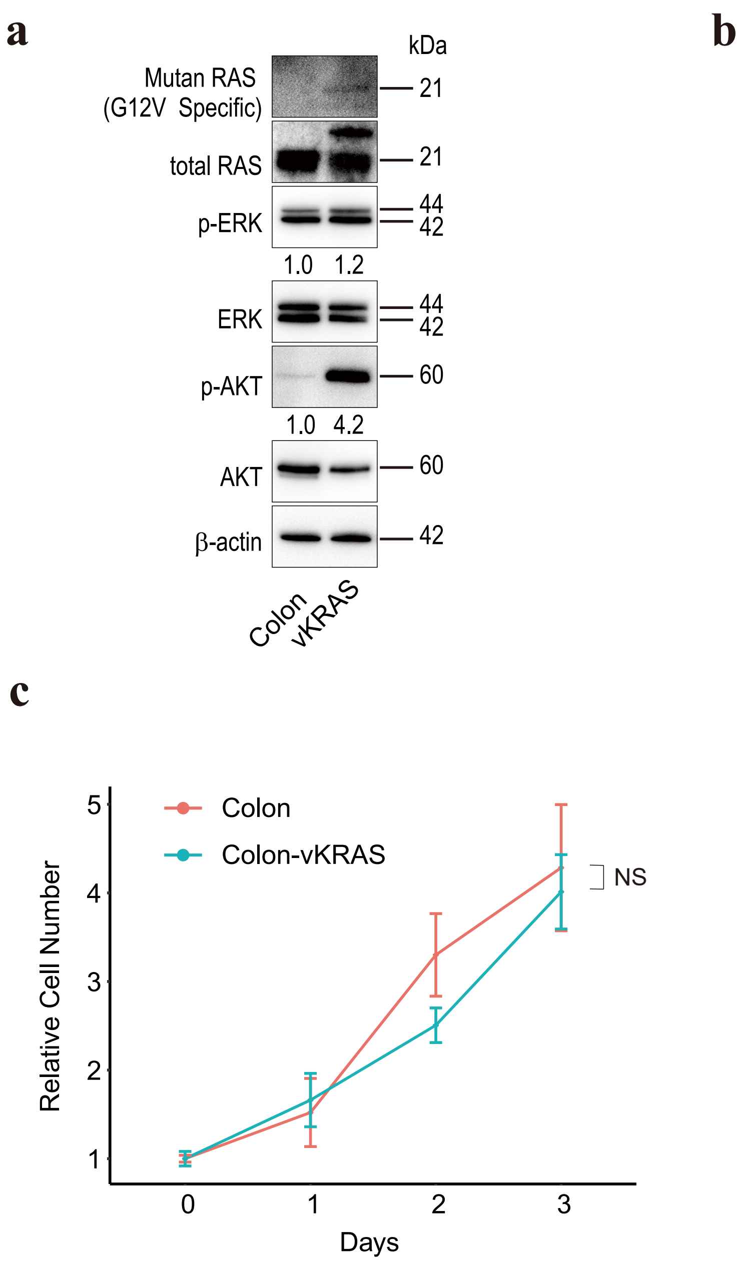

Supplementary Figure 3

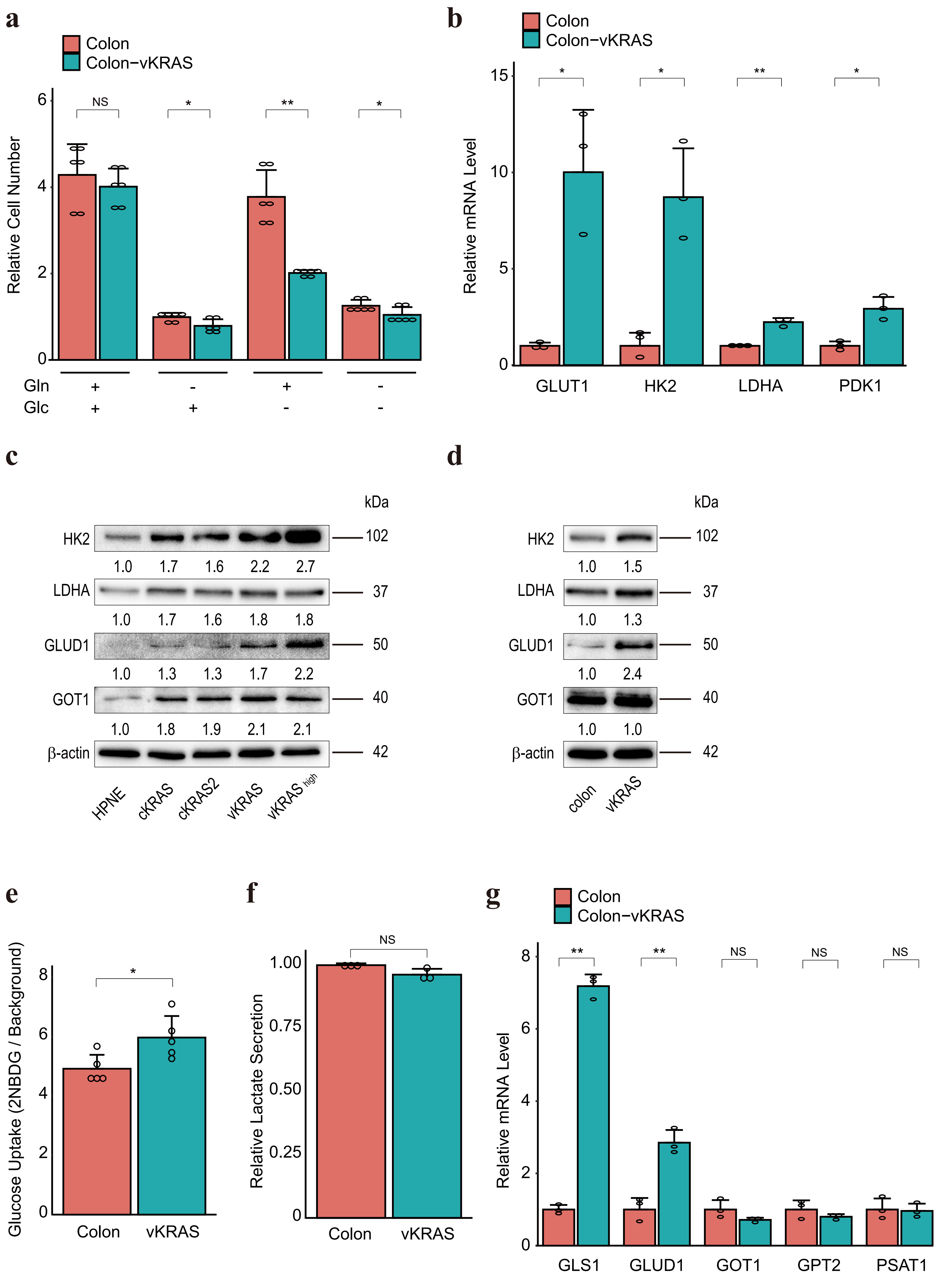

Supplementary Figure 4

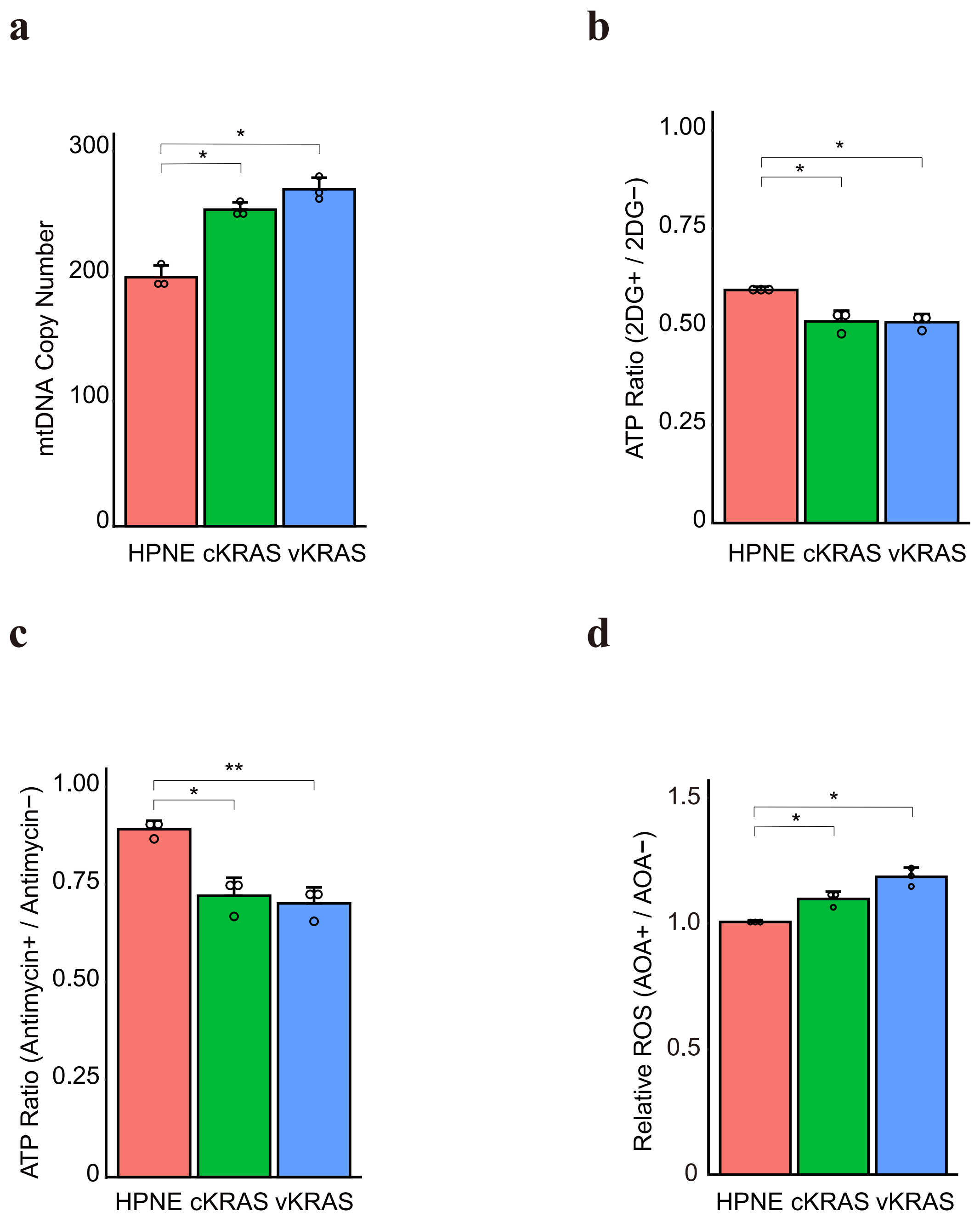

Supplementary Figure 5

a

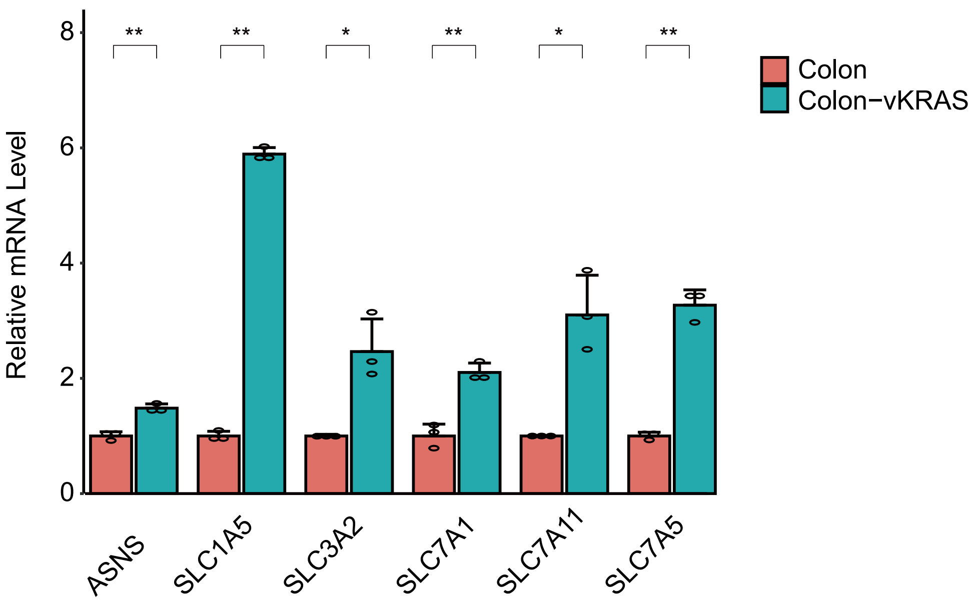

b

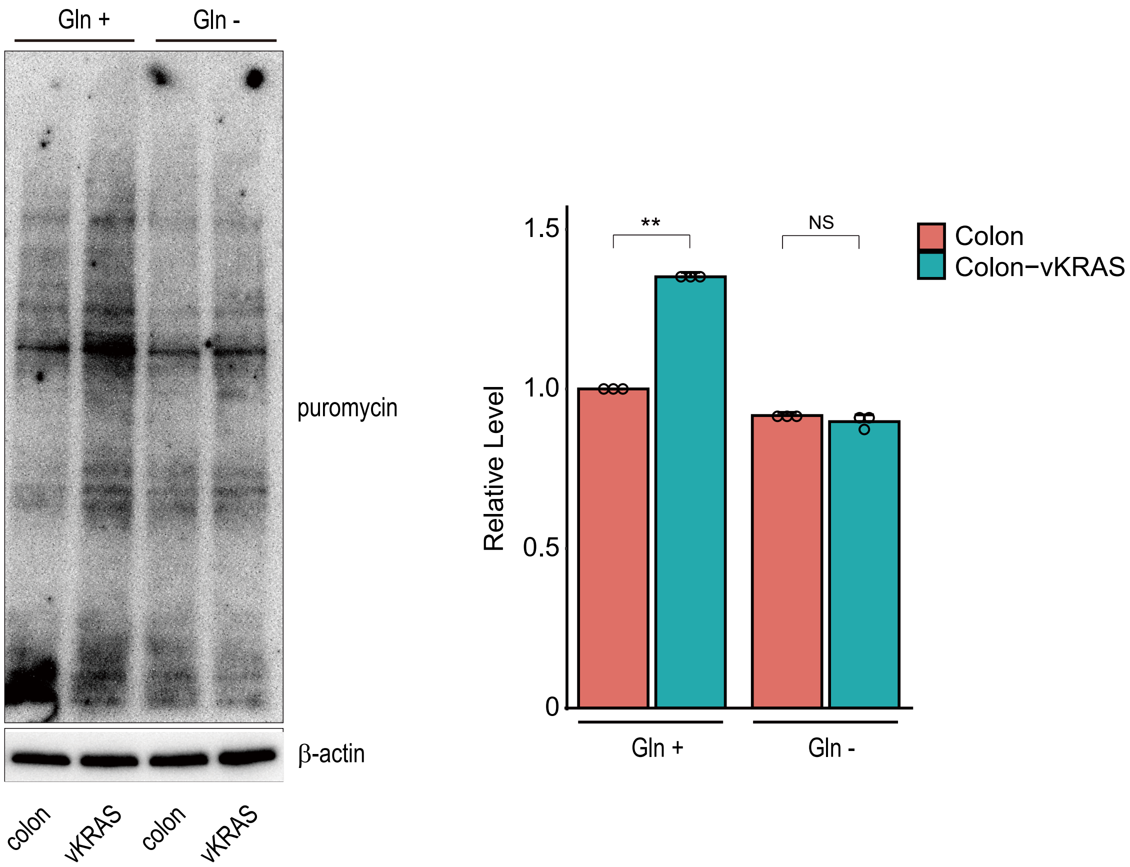

Supplementary Figure 6

a

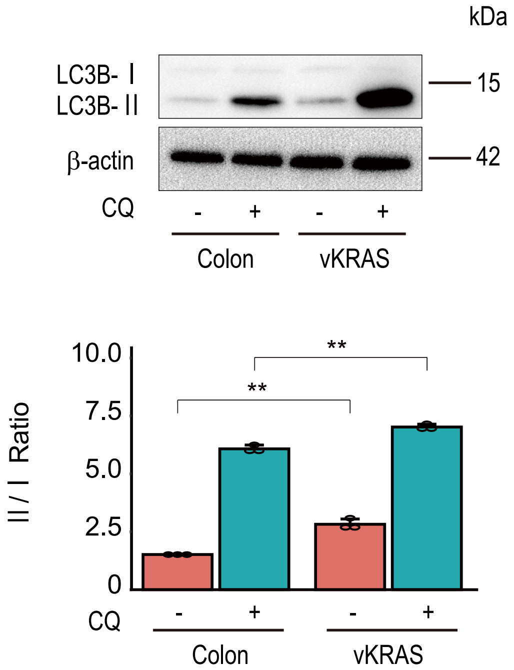

b

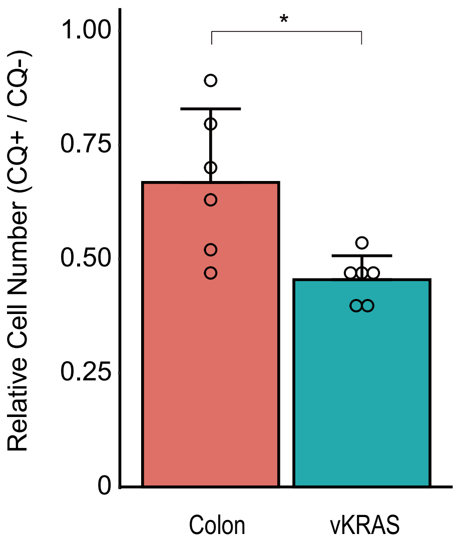

Supplementary Figure 7

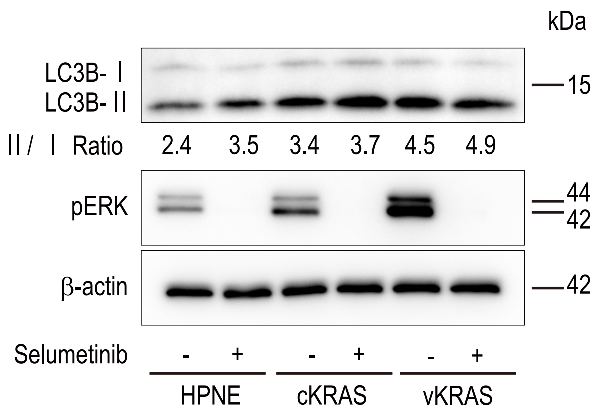

Supplementary Figure 8

Figure 1b

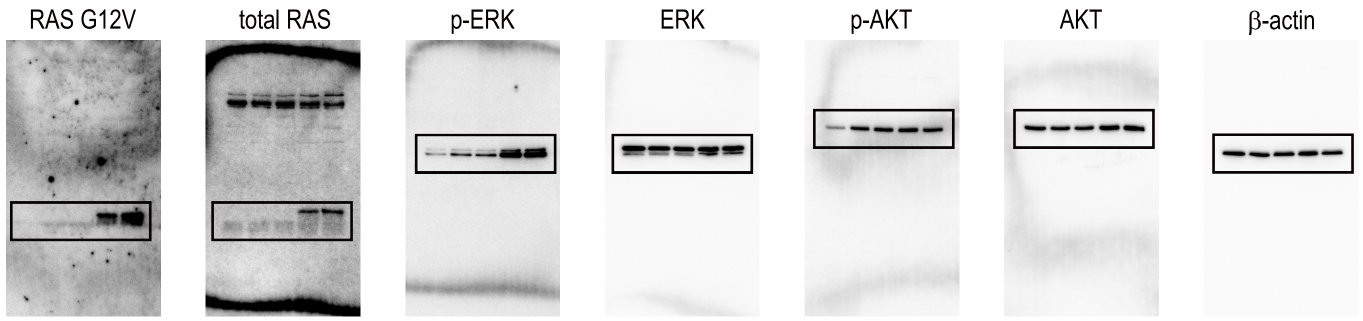

Figure 4d

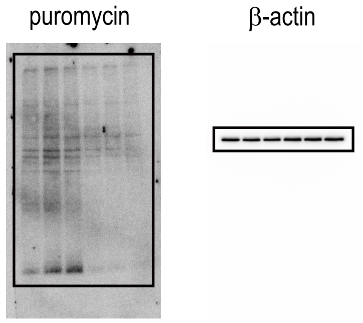

Figure 4e

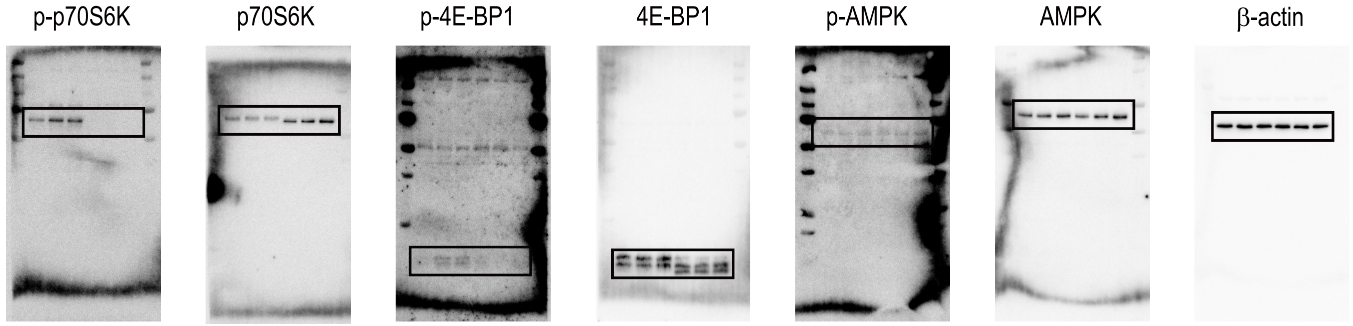

Figure 4f

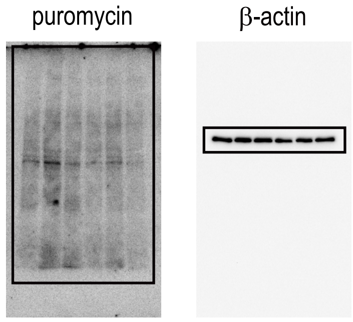

Figure 5a

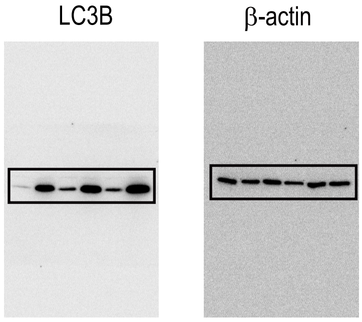

Figure 5b

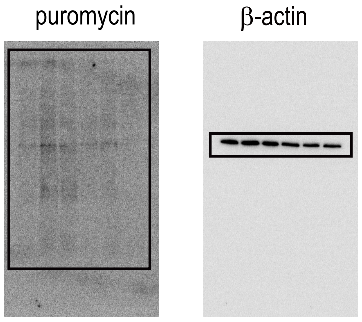

Supplementary Figure 9

Supplementary Figure 1f

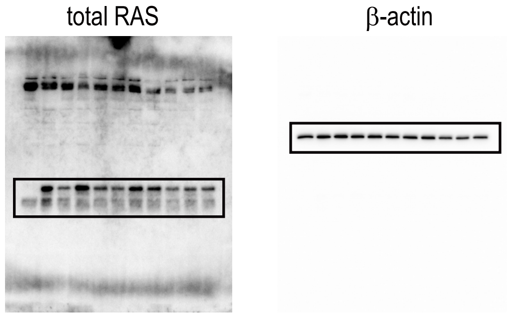

Supplementary Figure 2a

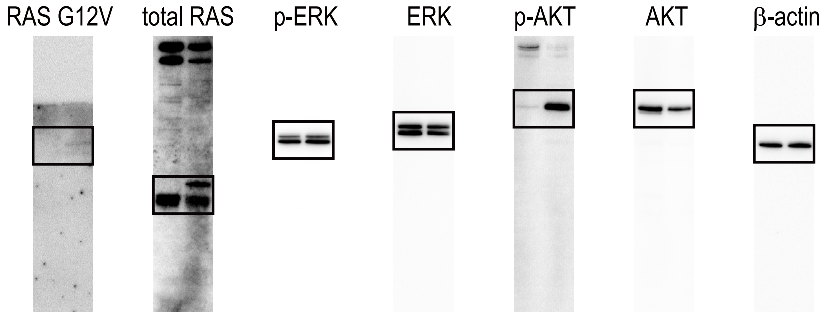

Supplementary Figure 3c

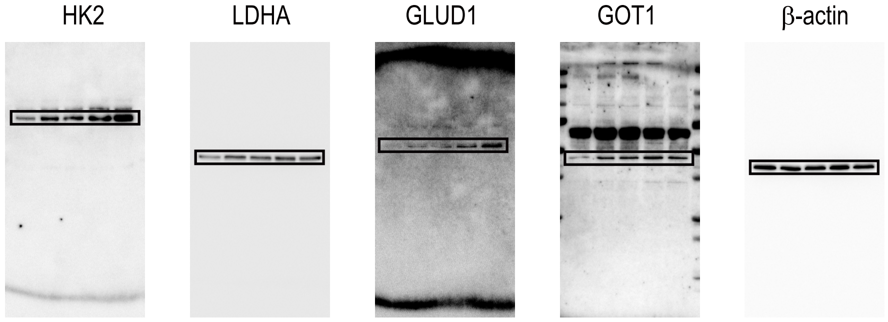

Supplementary Figure 3d

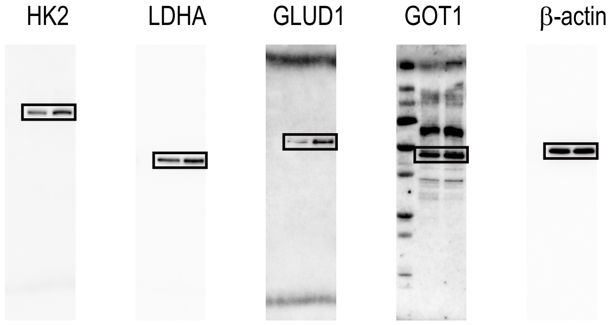

Supplementary Figure 5b

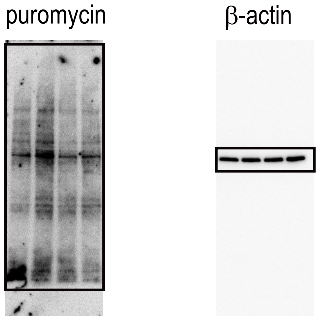

Supplementary Figure 6a

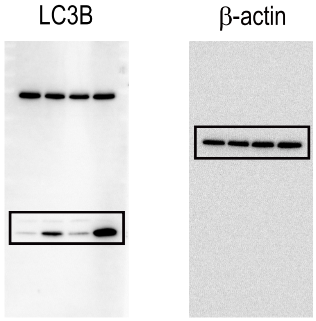

Supplementary Figure 7

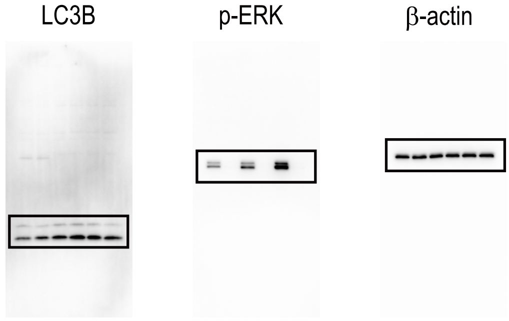

Supplement: Supplementary file 1 — Supplementary Information [file 41417_2021_326_MOESM1_ESM.pdf]
